# Role of Phytochemicals in Perturbation of Redox Homeostasis in Cancer

**DOI:** 10.3390/antiox10010083

**Published:** 2021-01-09

**Authors:** Shreyas Gaikwad, Sanjay K. Srivastava

**Affiliations:** Department of Immunotherapeutics and Biotechnology, Center for Tumor Immunology and Targeted Cancer Therapy, Texas Tech University Health Sciences Center, Abilene, TX 79601, USA; shreyas.gaikwad@ttuhsc.edu

**Keywords:** oxidative stress, cancer, reactive oxygen species (ROS), phytochemicals, dietary chemicals, natural compounds, programmed cell death, apoptosis, anoikis, autophagy, ferroptosis, pyroptosis

## Abstract

Over the past few decades, research on reactive oxygen species (ROS) has revealed their critical role in the initiation and progression of cancer by virtue of various transcription factors. At certain threshold values, ROS act as signaling molecules leading to activation of oncogenic pathways. However, if perturbated beyond the threshold values, ROS act in an anti-tumor manner leading to cellular death. ROS mediate cellular death through various programmed cell death (PCD) approaches such as apoptosis, autophagy, ferroptosis, etc. Thus, external stimulation of ROS beyond a threshold is considered a promising therapeutic strategy. Phytochemicals have been widely regarded as favorable therapeutic options in many diseased conditions. Over the past few decades, mechanistic studies on phytochemicals have revealed their effect on ROS homeostasis in cancer. Considering their favorable side effect profile, phytochemicals remain attractive treatment options in cancer. Herein, we review some of the most recent studies performed using phytochemicals and, we further delve into the mechanism of action enacted by individual phytochemicals for PCD in cancer.

## 1. Introduction

### 1.1. ROS and Its Physiological Role

Reactive oxygen species (ROS) is a blanket term which encompasses several reactive species derived from molecular oxygen [1]. ROS are short-lived highly reactive byproducts of aerobic metabolism. They are formed due to single-electron reduction of oxygen (O_2_) which forms radical superoxide (O_2_^•−^). The superoxide molecules are then converted to hydrogen peroxide (H_2_O_2_) in the presence of superoxide dismutase [2]. Reactions involving H_2_O_2_ and O_2_^−^ result in further generation of other ROS molecules such as hydroxyl radicals (OH), singlet oxygen (^1^O_2_), and peroxynitrite (ONOO^−^) [3]. Mitochondria is the biggest source of ROS where 80% of the ROS are generated. Once generated, ROS oxidize several cellular molecules and hence play diverse roles in various physiological processes of the cells [4]. ROS react with carbohydrates, lipids, proteins, and nucleic acids, resulting in functional alterations of pathways associated with cellular differentiation, proliferation, cytoskeletal regulation, and apoptosis [3,5]. Several protein structures such as ion channels, kinases, phosphatases are regulated by redox signaling [5,6]. In signal transduction, ROS transiently oxidizes the cysteine sulfhydryl group which is involved in the activation of most phosphatases [7]. ROS have been considered as a harmful by-product of aerobic metabolism, however, under balanced conditions, ROS play an important role as mediators of signaling pathways in various physiological systems [8]. The biological activity of ROS depends on the extent, location, and duration of action of the specific ROS molecule [9]. For example, in embryonic development, optimum levels of H_2_O_2_ are considered to be crucial for neuronal growth. A level of 1–10 nM H_2_O_2_ is required for neuronal growth as levels below 1 nM have shown spatial memory defects in mice studies, while levels above 10 nM result in axonal degeneration [10,11] In the cardiovascular system, mitochondrial ROS (mROS) are suggested to be involved in the dilation of human coronary resistance arteries in response to shear stress [12]. Additionally, in terms of optimal vascular angiogenesis, NADPH oxidase (NOX) is activated by vascular endothelial growth factor (VEGF) to produce ROS. The produced ROS and VEGF together then induce endothelial cell proliferation and migration [13]. For optimal blood vessel growth, a specific range of ROS called the “redox window” is required [14]. ROS have been observed to be involved in cognitive functioning wherein it was determined that mROS potentiates post-synaptic inhibitory signaling in cerebellar stellate cells via selective recruitment of α3-containing GABAA receptors [15]. Host-defense against pathogens was the first function of ROS to be discovered. Hypochlorous acid (HOCl) produced from chlorine and H_2_O_2_ generates chloramines which have cytotoxic effects [16]. In terms of host defense mechanism, after phagocytosis of the pathogen, NADPH oxidase (NOX) produces a high amount of ROS which in turn destructs the pathogen [17]. In the immune system, ROS coordinate the migration of polymorphonuclear leukocytes (PMNs) towards the pathogens, and then ROS also retain the PMNs at the site of infection [18]. T cell activation and proliferation are mediated through a signaling cascade mainly involving the MAPK/ERK pathway [19]. Protein tyrosine phosphatases (PTPs) negatively regulate the MAPK/ERK pathway and PTPs have a cysteine (Cys) residue in their active site which is regulated by ROS. ROS act as inhibitor of PTP by converting Cys residue in the active site to sulfenic acid (Cys-SOH), thus leading to activation of the MAPK/ERK pathway [20]. To summarize, ROS serve as important mediators in several physiological systems and the level of ROS molecules play a key role in its action.

### 1.2. Oxidative Stress in Cancer

A constant balance of ROS levels is required for appropriate homeostasis. Under normal conditions, our body’s antioxidant defense system continuously counteracts the oxidative attack from ROS molecules [21]. However, excessive accumulation of ROS within the cell disrupts the balance, leading to the generation of oxidative stress [22,23]. The generated oxidative stress leads to irreversible damage of DNA, peroxidation of lipids, oxidation of proteins, and inhibition of enzymes [1]. A high level of ROS present in oxidative stress conditions leads to the activation of various oncogenic pathways [6]. The phosphoinositide 3-kinases (PI3K) pathway is highly activated in a number of cancers. ROS primarily inactivate phosphatase and tensin homolog (PTEN) in the PI3K pathway by oxidizing Cys124, an active site on PTEN, leading to a disulfide bond with another intraprotein Cys71. Consequently, the inactivation of PTEN leads to hyper-activation of the PI3K pathway [24]. Constant phosphorylation of PI3K subsequently results in the activation of protein kinase B (AKT) via downstream pyruvate dehydrogenase kinase 1 (PDK1), ultimately leading to the upregulation of cell cycle stimulating genes such as proliferating cell nuclear antigen (PCNA) and cyclin-dependent kinase 1 (CDK1) [25]. In many Kirsten rat sarcoma viral oncogene homolog (KRAS) mutated oncogenic cancers, ROS play an important role in anchorage-independent growth through regulation of the ERK MAPK signaling pathway [26]. In the initial stage of tumor formation, angiogenesis is absent or poorly developed, resulting in hypoxic conditions. The hypoxic condition affects the mitochondrial electron transport chain (ETC), leading to increased ROS levels. Hypoxia-driven ROS play a role in the activation of hypoxia inducing factor-1 (HIF-1), a transcription factor that contributes towards glucose to lactate conversion for tumor glucose metabolism and the induction of VEGF. ROS activate the HIF-1 subunit by inactivating a HIF-1 inhibitor, PHD (prolyl dydroxylase domain) [27]. Increased ROS levels have been linked to tumor proliferation via the c-Myc pathway. At increased ROS levels, HIF-1 α dependent activation of c-Myc occurs, resulting in tumor proliferation and DNA damage [28]. ROS also play a role in tumor stromal environment and activate invasive tumor progression. Increased ROS levels in stromal fibroblasts by transforming growth factor beta-1 (TGFß1) initiated the mesenchymal–mesenchymal transition (MMT) and secretion of vascular endothelial growth factor (VEGF), hepatocyte growth factor (HGF), and interleukin-6 which are biomarkers for invasive tumor cells [29]. ROS affect the matrix metalloproteinase (MMP)/tissue inhibitor of metalloproteinase (TIMPs) ratio by activating MMP synthesis. ROS activate MMP synthesis either via Ras and MAPK signaling cascades or through NFκB pathway [30]. Cancer cells survive on levels of ROS that are slightly greater than normal cell counterparts and activate various oncogenic pathways. The cells adapt themselves to the moderate redox environment and proliferate. However, if ROS levels rise beyond a certain threshold, the transformed cancer cells are longer able to adapt and turn to various cell death pathways [31].

## 2. ROS and Associated Programmed Cell Death (PCD) Pathways

### 2.1. Apoptosis

Among all the PCD pathways, apoptosis is the most studied pathway which is mediated via a group of proteins called cysteine-dependent aspartate-directed proteases known as caspases [32]. ROS play a role in both extrinsic and intrinsic apoptosis pathways. The intrinsic pathway is mediated through the mitochondrial permeability transition pore (mPTP). ROS cause cytoplasmic release of cytochrome c from the mitochondria via its action on PTP. Specifically, ROS govern the regulation of three proteins adenine nucleotide translocase (ANT), cyclophilin D, and voltage-dependent anion-selective channel (VDAC) involved in the opening of PTP. ROS oxidizes specific cysteines in their active sites [33,34]. The intrinsic pathway is a more common apoptosis mode mediated by ROS since mitochondria is very sensitive to the increased ROS levels as it lacks DNA repair enzymes. In hyperactivated oxidative stress, the shutdown of mitochondrial function contributes to apoptosis as cellular energy supply stops [35,36]. In the extrinsic pathway also known as the death receptor pathway, TNF receptors are involved which are located at the plasma membrane and contain an intracellular death domain. These receptors recruit pro-caspases and adaptor proteins, which results in the formation of death-inducing signaling complex (DISC) and activates the caspases [37]. In this process, the cellular FLICE-inhibitory protein (c-FLIP) impedes the formation of DISC [38]. ROS mediate the downregulation of FLIP by ubiquitination and subsequent degradation by the proteasome. Primarily, H_2_O_2_ and O_2_^•−^ are responsible for FLIP down-regulation [39].

### 2.2. Autophagy

Autophagy is a catabolic process required for the regeneration of damaged organelles and cellular components under various stressful environments such as pathogen infection, growth factor deprivation, starvation, and intra-cellular stress [40]. Autophagy involves the generation of a double-membrane vesicle, called autophagosome, which engulfs the damaged components and merges with lysosome for hydrolytic degradation of the engulfed cargo [41,42]. A definite interplay exists between ROS and autophagy since both are induced by common oncogenic stimuli [43]. Under normal conditions, autophagy mediates cell survival by eliminating ROS and protecting the mitochondria. However, when stimulated, ROS induce excess autophagy resulting in cell death [44]. ROS regulates a negative regulator of autophagy, the mammalian target of rapamycin complex 1 (mTORC1). Inhibition of mTORC1 is mediated through cytoplasmic ataxia telangiectasia mutated (ATM). ATM activates the tuberous sclerosis complex 2 (TSC2) tumor suppressor which inhibits mTOR via stimulation of GTP hydrolysis of Ras family small GTPase Rheb (Ras homolog enriched in brain) [45,46]. Additionally, oxidative stress activates FOXO3 which stimulates the transcription of Bcl-2 nineteen kilodalton interacting protein (BNIP3) [47]. BNIP3 in turn competes with Beclin-1 for binding to Bcl-2. The unbound Beclin1 induce autophagic cell death [48]. However, autophagy acts in a context dependent manner and might play a pro-tumor role in certain conditions [49].

### 2.3. Ferroptosis

Ferroptosis, a novel form of programmed cell death, is characterized by the accumulation of iron and lipid peroxidation, leading to intracellular changes such as changes in mitochondrial structure and functioning [50]. The mechanism involved in ferroptosis is the suppression of system Xc, an amino acid antitransporter. System Xc is a part of an important antioxidant system in cells that provokes the synthesis of glutathione (GSH). GSH decreases ROS levels under the action of glutathione peroxidases (GPXs), thereby maintain a balanced oxidant-antioxidant environment. During ferroptosis, inhibiting system Xc causes decreased synthesis of GSH, leading to the accumulation of lipid ROS and ultimately, oxidative cell death [51]. Among GPXs, GPX4 is a pivotal player that counterbalances lipid peroxides and protects membrane integrity by using GSH as a cofactor [52]. In terms of cancer therapy, GPX4 is targeted as it is a specific and central regulator of ferroptotic cell death [53].

### 2.4. Pyroptosis

Pyroptosis is a caspase-dependent (caspase 1/4/5/11) mode of cell death, which is initiated by the formation of the inflammasome. The mechanism involves caspase-mediated cleavage of gasdermin D (GSDMD) and gasdermin E (GSDME). Following the cleavage, the N-terminal fragment of GSDMD is released and it forms pores in the cell membrane, leading to activation of inactive cytokines like IL-18 and IL-1β, water influx, cell swelling, and osmotic lysis [54]. ROS induce pyroptosis through the activation of NLR family pyrin domain containing 3 (NLRP3) inflammasome [55]. 

### 2.5. Anoikis

Anoikis is another apoptotic PCD wherein cells undergo death in the absence or improper attachment to the extracellular matrix (ECM). Anoikis is a Greek term for “homelessness” [56,57,58]. In cancer cells, the sonic hedgehog/Gli1 pathway is a well-known regulator of anoikis [59,60]. Additionally, ROS has been implicated to support anoikis resistance, however, depending upon the activation, ROS trigger anoikis-inducing cell death [61].

## 3. Phytochemicals: A Promising Role in ROS Mediated Cancer Cell Death

Considering the plethora of evidence generated from various studies, it is clear that beyond a threshold value, ROS play an anti-tumorigenic role. Therapeutic interventions to achieve the required threshold ROS level is a promising strategy for inducing PCD. Phytochemicals are naturally occurring compounds derived from plants and have been studied for their therapeutic effects in various physiological conditions. Multiple phytochemicals have proven their inheritance of cancer prevention and therapeutic properties [62,63]. The low side-effect profile of phytochemicals has made them a prominent arsenal against cancer. These naturally derived compounds are a valuable resource for cancer treatment and we hereupon discuss some of the phytochemicals studied in ROS-mediated anti-cancer activity.

### 3.1. Phytochemicals Acting via ROS-Mediated Apoptosis

#### 3.1.1. Capsaicin

Capsaicin (*N*-vanillyl-8-methylnonenamide) is a homovanillic acid derivative obtained from hot chili pepper [64]. Capsaicin has been widely studied for its anti-cancer activity [65,66]. Its activity is proposed to be mediated through oxidative stress-induced apoptosis. In pancreatic cancer cell lines BXPC-3 and AsPC-1, 4 to 6-fold ROS generation was observed within 1 h of capsaicin treatment while ROS generation was absent in normal pancreatic cells. Capsaicin induced oxidation of cardiolipin, a component of mitochondria. Oxidation of cardiolipin subsequently led to apoptosis. ETC complex-I and complex-III were observed to be the ROS-producing entities following capsaicin treatment [67,68].

#### 3.1.2. Sulforaphane

Sulforaphane (SFN; 1-isothiocyanato-4-(methyl-sulfinyl)-butane) is an isothiocyanate present in cruciferous vegetables like cabbage, broccoli, cauliflower, and kale [69]. Sulforaphane induced ROS mediated apoptosis in PC-3 and DU145 human prostate cancer cells. Specifically, caspase-8 activation and Fas protein induction following sulforaphane treatment confirmed apoptotic activity. ROS generation was suggested to be caused by both nonmitochondrial and mitochondrial mechanisms. GSH depletion was proposed to be the non-mitochondrial mechanism. ROS were postulated to be upstream activators of apoptosis based on caspase-8 attenuation following treatment with EUK-134 (SOD and catalase mimetic) [70].

#### 3.1.3. α-Lipoic Acid

α-lipoic acid (1,2-Dithiolan-3-yl pentanoic acid) is a naturally occurring dithiol compound found in fruits and vegetables [71]. In hepatocellular carcinoma cell line HepG2, it induced mitochondrial apoptosis preceded by ROS generation. Precisely, α-lipoic acid-induced ER (endoplasmic reticulum) stress wherein increased levels of C/EBP homologous protein (CHOP) were detected. ER stress led to activation of PERK (phospho- extracellular signal-related kinase) pathway and IRE1 (inositol-requiring enzyme 1) pathway leading to apoptosis. α-lipoic acid also inhibited ATF6 (activating transcription factor 6)-mediated pro-survival pathway [72].

#### 3.1.4. Benzyl Isothiocyanate (BITC)

BITC is an isothiocyanate present in cruciferous vegetables such as cauliflower, mustard, watercress, cabbage, and horseradish [73]. BITC has been studied for its anti-cancer effects in various studies previously [74,75,76,77,78]. BITC was studied for its anti-cancer potential in pancreatic cancer cell lines Capan-2 and MIAPaCa-2 cells. BITC treatment activated MAPK signaling with activation of ERK and JNK observed within as early as 1 h of treatment while ROS generation was observed within 30 min of treatment. Additionally, activation of MAPK family members was absent in normal HPDE-6 cell line. Thus, BITC treatment led to ROS generation followed by activation of MAPK family members ERK, JNK, and P38 and subsequently apoptosis [79].

#### 3.1.5. Phenethyl Isothiocyanate (PEITC)

PEITC, another isothiocyanate present in cruciferous vegetables has been studied for its anti-cancer effects and ROS-generating properties [80,81,82,83,84,85]. In a study to investigate effects of PEITC on breast cancer cells, PEITC showed ROS-mediated apoptosis in MDA-MB-231 and MCF-7 cell lines. High levels of hydrogen peroxide were observed in the cancer cells following treatment with PEITC. An antioxidant pre-treatment blocked cellular death and percent apoptosis induced by PEITC. Thus, confirming high levels of ROS led to apoptotic cell death [86].

#### 3.1.6. Piperine

Piperine [1-(5-[1,3-benzodioxol-5-yl]-1-oxo-2,4-pentadienyl) piperidine] is an alkaloid extracted from black pepper (*P. nigrum*) and long pepper (*P. longum*) [87]. Various small molecules derived from the *Piperaceae* family have been implicated in ROS-mediated apoptotic cell death of cancer cells [88,89]. Piperine has been implicated for ROS generation in SKMEL-28 and B16-F0 melanoma cell lines. ROS generation was observed within 30 min of treatment and the levels sustained until 24 h. Phosphorylation of Chk1 (checkpoint kinase 1) was observed in response to DNA damage which consequently led to G1 cell cycle arrest and apoptosis. Significant expression of phosphorylated ataxia telangiectasia and Rad3 related protein (ATR) at Ser 428 and Chk1 at Ser 296 was observed, suggesting DNA damage [90].

#### 3.1.7. Curcumin

**Figure 1:**
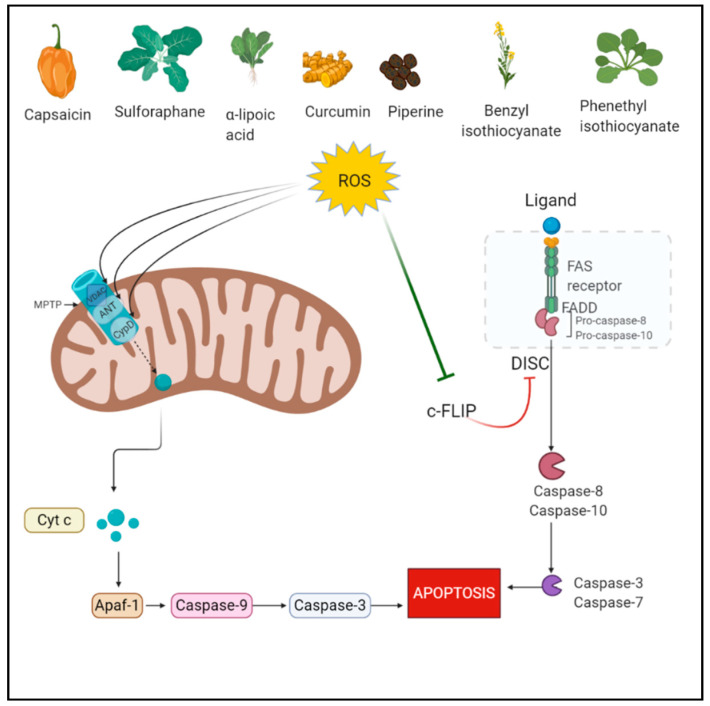
Reactive Oxygen Species (ROS)-mediated apoptotic action of phytochemicals. Figure created with BioRender.com.

Curcumin (1,7-bis(4-hydroxy-3-methoxyphenyl)-1,6-heptadiene-3,5-dione) is a polyphenol derived from rhizome of Curcuma longa. It possesses pleiotropic therapeutic benefits including anti-cancer effects [91,92]. The apoptotic role of curcumin was studied in colorectal cancer and sequential dosing was considered important for ROS-mediated apoptotic induction. In the study, the sequential treatment caused lysosomal permeabilization and ROS generation, accompanied by autophagic dysregulation and ER stress. Lysosomal permeabilization resulted in BID-dependent mitochondrial membrane permeabilization and thereby caspase-dependent apoptosis. Cathepsin B was confirmed to be involved in apoptosis following sequential treatment [93]. Figure 1 depicts the ROS-mediated apoptotic action of phytochemicals.

### 3.2. Phytochemicals Acting via ROS-Mediated Ferroptosis

#### 3.2.1. Withaferin A

Withaferin A (WA) is a steroidal lactone isolated from the plant *Withania somnifera*. It has been widely studied for anti-inflammatory and anti-tumorigenic properties [94]. Withaferin A induced ferroptotic cell death in neuroblastoma cells. WA targeted Keap-1 (kelch-like ECH-associated protein (1), a negative regulator of Nrf2 (nuclear factor erythroid 2-related factor (2). Keap-1 inhibition caused the release of Nrf2. The released Nrf2 upregulated HMOX1 [heme oxygenase (decycling) 1] which catalyzes heme degradation to generate ferrous iron (Fe^2+^). A high concentration of Fe^2+^ led to ROS generation and ultimately cellular death [95].

#### 3.2.2. Bromelain

Bromelain is a mixture of proteolytic enzymes derived from the stem of pineapple plants belonging to the *Bromeliaceae* family [96]. Bromelain was shown to induce ferroptotic cell death in KRAS-mutant colorectal cancer wherein bromelain increased the level of long-chain acyl-CoA synthetase-4 (ACSL4) an isozyme which plays role in lipid biosynthesis and fatty acid degradation. Specifically, ACSL4 accumulates oxidized cellular membrane phosphoplipids. ACSL4 mainly acts on phosphatidylethanolamine levels which act as an executioner of ferroptotic cell death [81,97].

#### 3.2.3. Ruscogenin

Ruscogenin [(1β,3β,25*R*)-Spirost-5-ene-1,3-diol] is a steroidal sapogenin extracted from *Ruscus aculeatus* plant [98]. Ruscogenin was studied for ferroptotic effects in pancreatic cancer cell lines BxPC-3, SW1990, PANC-1, and AsPC-1. Ruscogenin treatment increased Fe^2+^ ion concentration by upregulating the expression of transferrin and downregulating the expression of ferroportin. To further confirm the mode of action, cell death by ruscogenin reduced following treatment with iron chelator and cell death increased when ferric ammonium citrate was added. To conclude, ruscogenin regulated iron transport resulted in overproduction of ROS, leading to cell death [99].

#### 3.2.4. Oridonin

**Figure 2:**
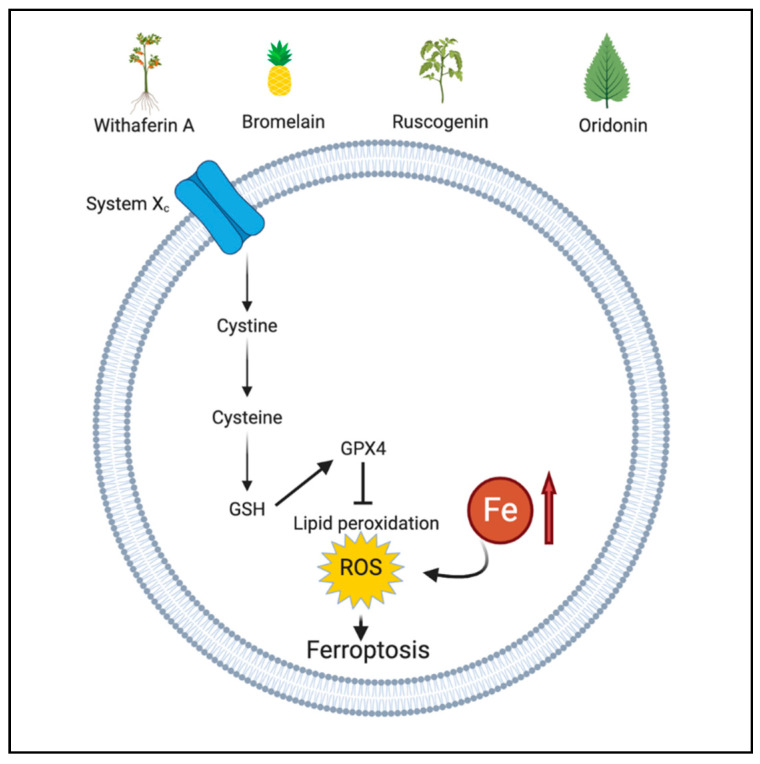
ROS-mediated ferroptotic action of phytochemicals. Figure created with BioRender.com.

Oridonin (Ori) is a tetracyclic diterpenoid obtained from *Isodon Rubescens* [100]. The ferroptotic effect of Ori was studied in esophageal cancer cell line TE1 and inhibition of the gamma-glutamyl cycle was observed in oridonin treated cells. Precisely, Ori affected GGT1, a key enzyme in the gamma-glutamyl cycle, which is crucial for protecting cells from oxidative stress and maintaining cysteine homeostasis. Additionally, Ori inhibited GSH by a covalently binding with cysteine after entering the TE1 cells. The covalent binding reduces intracellular cysteine levels which is responsible for ferroptosis. A decrease in GPX4 level was also detected after Ori treatment [101,102]. Figure 2 depicts the ROS-mediated ferroptotic action of phytochemicals.

### 3.3. Phytochemicals Acting via ROS-Mediated Autophagy

#### 3.3.1. Cucurbitacin B

Cucurbitacin B (1,2-Dihydro-alpha-elaterin) is a tetracyclic triterpene compound found in the plants of *Cucurbitaceae* and other also a variety of other plant families. Some of the sources of cucurbitacin B are *Helicteres angustifolia*, *Licaniaintra petiolaris*, *Casearia arborea*, and *Cucumis prophetarum* [103]. Cucurbitacin B has been studied for its anticancer effects in human cervical cancer HeLa cells. Cucurbitacin B induced autophagic cell death and was observed to have mediated through mitochondrial ROS production. Treatment of HeLa cells with cucurbitacin-B resulted in accumulation of several lamellar structures having cytosolic autophagic vacuoles. Additionally, the conversion of non-autophagic soluble LC3 (LC3-I) to autophagic LC3 (LC3-II) was detected following treatment with cucurbitacin-B. Pretreatment with antioxidant NAC and Mito-TEMPO (mitochondria-targeting antioxidant) inhibited LC3-II conversion, cell death, and autophagosome formation. Thus, the results indicated that ROS plays an important role in promoting autophagic cell death following cucurbitacin treatment [104]. Another study confirmed cucurbitacin-B induced autophagic cell death via ROS in MCF-7 breast cancer cells. The role of ROS was confirmed by the use of antioxidant pretreatment which reduced protein expression of LC3 II [105]. 

#### 3.3.2. Silibinin

Silibinin is a flavonoid derived from the milk thistle plant (*Silybum marianum*). The autophagic activity of silibinin was studied in human fibro-sarcoma HT1080 cells wherein silibinin was shown to activate p53 via the ROS-p38 pathway. Activation of p53 partially mediated autophagic cell death by inhibiting the MEK/ERK1/2 and PI3K/Akt pathway. An increased expression of Beclin1 (autophagosome marker) and conversion of LC3 I to LC3 II was observed following silibinin treatment [106]. Another study indicated that silibinin induces autophagic cell death mediated through mitochondrial dysfunction [107]. 

#### 3.3.3. Allicin

Allicin (2-propene-1-sulfinothioic acid *S*-2-propenyl ester) is an organosulfur compound present in garlic initially studied for its anti-bacterial studies [108]. Allicin was shown to induce both autophagy and apoptosis via increased production of ROS in non-small cell lung cancer cell lines A549 (adenocarcinoma) and NCI-H460 (large cell carcinoma). The authors observed a dose-dependent autophagic action of allicin. At low doses, allicin induced autophagy associated with moderate ROS levels, while increased doses resulted in higher ROS production and lysosomal disruption leading to cell death. The lysosomal disruption resulted in intra-cellular hydrolytic enzyme release [109]. Similar autophagic action of allicin was studied in hepatocellular carcinoma cells. Interestingly, allicin caused autophagy induction in p53 normal human liver cancer cells while apoptosis in p53 deficient cell line showing the dependence of its autophagic action on p53 [110].

#### 3.3.4. Carnosol

Carnosol is an ortho-diphenolic diterpene found in Mediterranean herbs rosemary, sage, and oregano [111]. Although it has been studied for its antioxidant activity, some evidence also shows the cytotoxic effects of carnosol via ROS overproduction. ROS production by carnosol is a dose and time-dependent process [112]. Carnosol study in MDA-MB 231 breast cancer cells showed ROS accumulation within 1 h of treatment while LC3II accumulation at 3 h post-treatment, suggesting ROS to be an upstream inducer of autophagy. It was observed that at lower concentration (<25 µM), carnosol-induced ROS-mediated autophagy only, while at higher concentration (>50 µM), ROS induced both autophagy as well as apoptosis. Interestingly, autophagy induction by carnosol was beclin-1 independent [113].

#### 3.3.5. Quercetin

Quercetin is a flavonoid found in fruits such as grapes, red raspberry, apples, and vegetables like broccoli, shallots, tomatoes, onion, and tea [114]. Quercetin has been widely studied for its anti-cancer effects and exerts its effects through multiple pathways. A study of quercetin in osteosarcoma cells revealed it activates autophagic flux via its action on nuclear protein 1 (NUPR1) which is involved in autophagosome formation, cargo degradation, and autophagosome-lysosome fusion. It was observed that quercetin-induced overproduction of ROS led to increased expression and activation of NUPR1. Although a majority of the studies show an inverse relation between NUPR1 and ROS, a direct relation is hypothesized [115,116].

#### 3.3.6. Berberine

**Figure 3:**
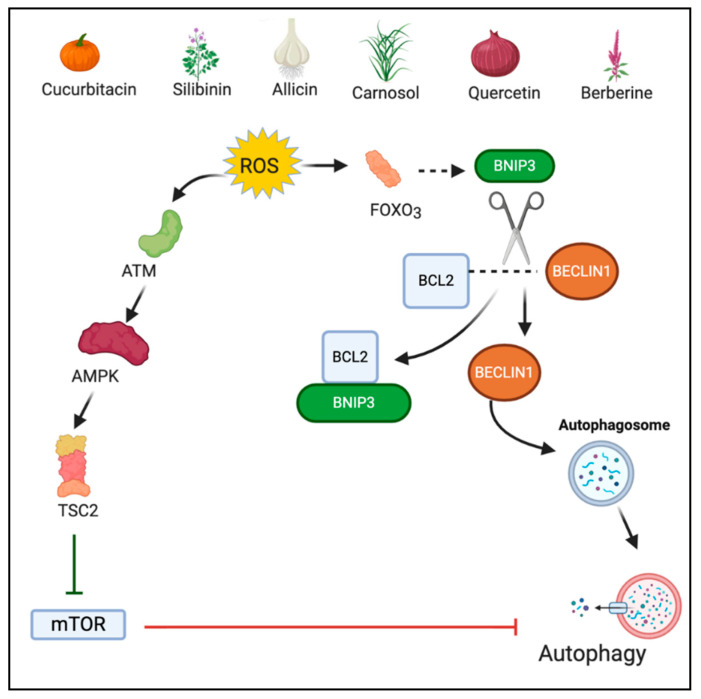
ROS-mediated autophagic action of phytochemicals. Figure created with BioRender.com.

Berberine (5,6-dihydro-9,10-dimethoxybenzo[g]-1,3-benzodioxolo[5,6-a] quinolizinium) is a quaternary benzylisoquinoline alkaloid derived from stem bark and roots of many plants and mainly the plants belonging to genus *Berberis* [117]. Recently, berberine was investigated for its anticancer role in the renal cell carcinoma model wherein it was combined with photodynamic therapy (PDT). The fluorescent nature of berberine was a key characteristic of the combinational effect. Berberine was used as a photosensitizer agent because the complementary action of a photosensitizer agent with a laser induces ROS generation, leading to cellular death. Berberine along with PDT showed significantly increased autophagy in comparison to berberine alone, suggesting photoactivation of berberine is required for ROS-mediated autophagy and ultimate cell death [118]. Figure 3 depicts the ROS-mediated autophagic action of phytochemicals.

### 3.4. Phytochemicals Acting via ROS-Mediated Pyroptosis

#### Nobiletin

**Figure 4:**
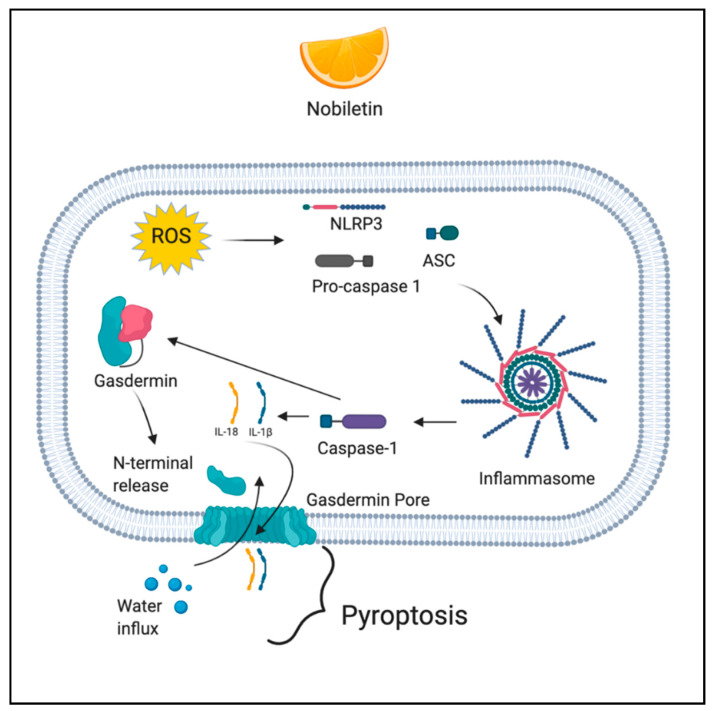
ROS-mediated pyroptotic action of phytochemicals. Figure created with BioRender.com.

Nobiletin (5,6,7,8,3′,4′-hexamethoxyflavone) is a polymethoxyflavonoid extracted from citrus fruits. Nobiletin has various pharmacological activities such as antimetabolic disorder, anticancer, neuroprotection, anti-inflammation, antioxidation, and cardiovascular protection [119]. Nobiletin was studied for its anti-cancer activity in human ovarian carcinoma cell lines A2780 and OVCAR3. In the study, nobiletin treatment resulted in ROS generation, exhibited by a significant decrease in mitochondrial membrane potential in a dose-dependent manner. Further, ROS contributed to the cleavage of GSDMD and GSDME, the two biomarkers of pyroptosis among which GSDMD is considered to be an executioner of pyroptosis [120,121]. Figure 4 depicts the ROS-mediated pyroptotic action of phytochemicals.

### 3.5. Phytochemicals Acting via ROS-Mediated Anoikis

#### Emodin

**Figure 5:**
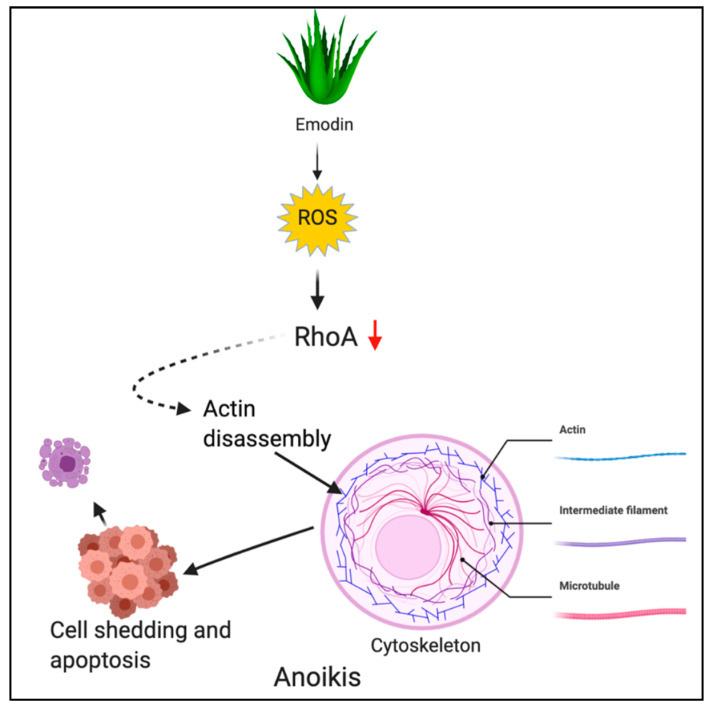
ROS-mediated anoikis by phytochemicals. Figure created with BioRender.com.

Emodin (1,3,8-trihydroxy-6-methylanthraquinone) is an anthraquinone derivative obtained from rhubarb plant (*Rheum palmatum*) and *Aloe vera* [122]. Emodin was shown to induce ROS generation followed by anoikis in gastric cancer cells. Emodin induced oxidative stress and caused inactivation of RhoA, a crucial signaling molecule in the cytoskeletal rearrangement. Inactivation of RhoA led to the disruption of focal adhesion complex and ultimately anoikis [61]. Figure 5 depicts the ROS-mediated anoikis by phytochemicals.

## 4. Conclusions

The ability of natural phytochemicals to modulate various signaling pathways make them promising therapeutic candidates in cancer. Many phytochemicals have progressed towards clinical trials against various malignancies such as breast, pancreatic, colon, and prostate cancers where promising results have been observed. Since cancer cells survive on levels of ROS that are slightly higher than normal cells, they are more sensitive to external perturbation. Fine-tuning of ROS levels using phytochemicals is a promising therapeutic strategy in cancer. Additionally, recent findings such as photooxidative stress using phytochemicals have provided selective targeting of cancer cells. However, there are a few challenges which need to be addressed in terms of the pharmacokinetic parameters of phytochemicals. In order to induce sufficient ROS levels for anti-cancer activity, specific plasma levels of the phytochemicals need to be attained. It has been observed that some of the phytochemicals do achieve the required plasma levels. A few such candidates having good pharmacokinetic parameters include BITC, PEITC, silibinin, and berberine. However, the majority of the phytochemicals fail to achieve required cytotoxic levels. This issue can be addressed by formulating the natural compound in advanced drug delivery systems such as nano drug delivery formulations. Various studies have been conducted in this aspect and nano drug delivery has proven to be an effective tool to achieve high plasma concentrations of the phytochemicals. Some of the successful candidates include sulforaphane and curcumin. A nano structured lipid formulation increased the bioavalability of sulforaphane in rat plasma while a nanoparticle formulation increased curcumin levels in human plasma by 10–15-fold. In the future, large cohort studies of ROS targeted phytochemicals are required, since these studies will provide data regarding efficacy, toxicity, bioavailability in clinical setup for their establishment as anti-cancer therapies in the market. An outline of pharmacokinetic data, selective action between normal and cancer cells by phytochemicals has been presented in Table 1.

## Figures and Tables

**Table 1:** Phytochemicals are associated with ROS-mediated programmed cell death in cancer.

Sr. No.	Phytochemical	PCD Type	Effective Anti-Cancer Concentration (In Vitro)	Dose Administered (In Vivo)	Pharmacokinetic Data	TttCell Lines/Subjects	Outcome/Comments	References
1	Capsaicin	Apoptosis	150 µM	2.5 mg/kg	Not stated	Animal subject—Mice In vitro study Pancreatic cancer cells	Apoptosis was observed in AsPC-1 and BXPC_3_ cell lines at 150 µM concentration, while no effect were observed in normal (HPDE-6) cells. In an animal study, an oral dose 2.5 mg/kg was effective in suppressing tumor growth. Human equivalent dose—0.202 mg/kg.	[67,68]
2	Capsaicin (Pharmacokinetic study)	-	-	5 g	C_max_: 2.47 ± 0.13 ng/mL	Human subjects	The oral bioavailability of capsaicin is effective for anti-diabetic effect, but plasma level is less for its anti-cancer activity.	[123,124]
3	Sulforaphane	Apoptosis	5–10 µM	-	-	-	Various studies report anticancer activity in a range of 5–10 µM which is not attainable through dietary intake. A study in normal vs. cancerous prostate cells revealed selective cytotoxicity of sulforaphane in cancerous cells. No effect was observed in normal cells at a dose range of 0–15 µM.	[125,126]
4	Sulforaphane (Pharmacokinetic study)	-	-	30 mg/kg	C_max_: 772.8 ± 54.36 ng/mL	Animal subject—Rats	Nano structured lipid formulation of sulforaphane increased its bioavailability in rat plasma wherein a dose close to effective anticancer level was achieved.	[127]
5	α-lipoic acid	Apoptosis	500 µM	-	-	In vitro study hepatoma cell line	ROS-mediated apoptosis was observed at 500 µM. However, in another study, no effect was observed in normal L02 liver cells at 5 mM concentration.	[72,128]
6	α-lipoic acid (Pharmacokinetic study)	-	-	600 mg per day	C_max_: 8–52 nM	Human subjects	Plasma levels achieved in plasma following oral administration of racemic α-lipoic acid is much less than required for its anti-cancer activity.	[129]
7	BITC	Apoptosis	-	-	-	In vitro study Pancreatic cancer cells	BITC caused ROS generation in a concentration dependent manner starting from 2.5 to 20 µM in MIAPaCa-2 and Capan-2 cell lines	[79,130]
8	BITC	Apoptosis	<2.5–5 µM	-	-	In vitro study Breast cancer cells and normal mammary epithelial cell line	BITC induced ROS-mediated apoptosis in MDA-MB-231 and MCF-7 at IC_50_ value of <2.5 µm and 5 µM respectively at 24-h time point. However, the IC_50_ was 20 µM in normal mammary epithelial cell line MCF-10A.	[131]
9	BITC	Apoptosis	8 µM	-	-	In vitro study Pancreatic cancer cells	An IC_50_ of 8 µM was observed when BXPC_3_ cells were treated with BITC.	[132]
10	BITC (Pharmacokinetic study)	-	-	12 µM/day	C_max_: 7.5 µM in tumor	Animal subject—Mice	BXPC3 cells were subcutaneously implanted and a 43% tumor inhibition was observed with a concentration near IC_50_ value achieved in plasma.	[74]
11	PEITC	Apoptosis	<5 µM	-	-	In vitro study Breast cancer cells	PEITC caused ROS-mediated apoptosis in MDA-MB-231 and MCF-7 cell lines.	[86]
12	PEITC	Apoptosis	5–10 µM	-	-	In vitro study normal ovarian epithelial cell line	Ovarian epithelial cell line T72 was transfected to over express *Ras* oncogene to form oncogenic T72Ras cell line. Following PEITC treatment, the transformed cells showed higher sensitivity to ROS as compared to normal cells. Thus, showing selective activity of PEITC.	[133]
13	PEITC (Pharmacokinetic study)	-	-	10–100 µmol/kg	C_max_: 9–42 µM	Animal subjects—Rats	A considerably high level of PEITC was achieved after oral administration of PEITC than required for its anti-cancer activity.	[134]
14	Piperine	Apoptosis	136–137 µM	-	-	In vitro study Melanoma cell lines	Piperine induced RPS mediated apoptosis in SKMEL-28 and B16-F0 cells at 136 µM and 137 µM respectively at 72-h time point.	[90]
15	Piperine	Apoptosis	132 µg/mL	-	-	In vitro study Lung cancer cells and normal lung fibroblasts	Piperine induced apoptotic cell death via p53 dependent mitochondrial pathway in A549 cancer cell line while no significant cytotoxicity was observed in normal WI38 human lung fibroblasts. ROS are considered to be downstream effectors of p53 mediated apoptosis.	[135,136]
16	Piperine (Pharmacokinetic study)	-	-	200 mg tablets (Benjakul formulation)	C_max_: 1.078 µg/mL	Human subjects	Piperine is a major component of a traditional Thai medication called Benjakul. A dose of 200mg resulted in a Cmax level of 1.078 µg/mL which is significantly lower than that required for anticancer effect.	[137]
17	Curcumin	Apoptosis	25 µM	5 mg/kg	-	In vitro study Colorectal cancer cells Animal subjects—Mice	Curcumin induced ROS-mediated apoptotic cell death in HCT-116 cell line at 25 µM concentration within 48hh. Curcumin showed considerable tumor inhibition in in vivo xenograft model following administration of 5 mg/kg I.P dose.	[93]
18	Curcumin	Apoptosis	20 µM	-	-	In vitro study Cervical cancer cells and normal cervical cells	Curcumin induced ROS-mediated apoptosis in cervical cancer cell lines C33A, CaSki, HeLa, and ME180 cells at approximately 20 µM in 48-h time period, while it did not induce significant toxicity in normal counterparts until 40 µM concentration.	[138]
19	Curcumin (Pharmacokinetic study)	-	-	Various doses (2–10 g)	C_max_: 1–3200 ng/mL	Healthy human volunteers and patients (cancer, Alzheimer’s disease etc.)	The serum levels of curcumin achieved by oral administration of crude curcumin are much lower than required for anti-cancer activity. However, a 10–15-fold increase in plasma levels was observed when curcumin was formulated as nanoparticle or combined with piperine, lecithin, etc.	[139]
20	Withaferin A	Ferroptosis	5–10 µM	4 mg/kg	Not stated	In vitro study Neuroblastoma cell lines Animal subjects— Mice	Crude Withaferin A and its nano particle formulation for tumor targeting showed ROS-mediated ferroptosis at 10 µM in neuroblastoma cell lines IMR-32, SK-N-SH, Kelly, NB69, and CHP-134 within 4–8 h. Tumor regression was mediated through lipid peroxidation.	[95]
21	Withaferin A	Apoptotic	1–50 µM	4 mg/kg	C_max_: 1.8 µM	In vitro study Breast cancer cell lines Animal subject—Mice	Withaferin A displayed cytotoxicity in breast carcinoma cell lines MDA-MB 231, H1299, T47D, MCF-7, LN686, as well as normal fibroblast cell line COS-7 in a wide range of 1–50 µM at 72-h time point. The IC_50_ was lesser than normal fibroblasts in majority of the cancer cell lines.	[140]
22	Bromelain	Ferroptosis	-	3 mg/kg	Not stated	In vitro study Colon carcinoma cell lines and normal colon cell line. Animal subjects—Mice	Bromelain inhibited proliferation of in Kras mutant colon cancer cell lines HCT-116 and DLD-1 at 50 µg/mL, while it induced significant ferroptosis in cancer cell lines at a concentration of 5 µm when combined with Erastin as compared to normal colon cells CCD1co. Bromelain increased survival rate in treatment group as compared to vehicle group.	[81]
23	Bromelain (Pharmacokinetic study)	-	-	143 mg/kg	C_max_: Very low (specific value not stated)	Human subjects	Bromelain showed very low (ng/mL) plasma levels following oral administration at 143 mg/kg body weight.	[141]
24	Ruscogenin	Ferroptosis	7.3–28.19 µM	5 or 10 mg/kg	Not stated	In vitro study Pancreatic cancer cells Animal subjects—Mice	Ruscogenin induced significant ferroptotic cell death in pancreatic cancer cell lines BxPC-3, SW1990, PANC-1, and ASPC-1 cells, as compared to normal pancreatic cell line HPDE-6-C7 wherein not IC_50_ was detected at 72-h time point in HPDE6-C7 cells. Additionally, it inhibited pancreatic cancer growth in vivo.	[99]
25	Ruscogenin (Pharmacokinetic study)	-	-	8 mg/kg	C_max_: 504.50 ± 63.47 ng/mL (Mean value)	Animal subjects—Rats	Following IV administration of Ruscogenin in rats, a plasma level between 2–1000 ng/mL was observed which roughly translates to 2.35 µM, a level much lower than required for anticancer effect.	[142]
26	Oridonin	Ferroptosis	26.93 µM	-	-	In vitro study Esophageal cancer cells	Oridonin induced ferroptotic cell death in esophageal cancer cell line TE1 at 27 µM within 24-h time point.	[101]
27	Oridonin	Apoptosis	2.5–10 µM	-	-	In vitro study Esophageal cancer cell lines	Oridonin induced cellular death in human esophageal cancer cell lines (KYSE70, KYSE410, and KYSE450) at 2.5–10 µM concentration, while it showed 40% cell death in normal esophageal cell line SHEE at 40 µM, a significantly higher concentration.	[143]
28	Oridonin (Pharmacokinetic study)	-	-	20 mg/kg	C_max_: 146.9 ± 10.17 ng/mL	Animal subjects—Rats	A concentration of approximately 150 ng/mL translating to 412 nM was achieved. Combination with verapamil increased the Cmax level of oridonin to 194 ± 10 ng/mL, still a lower level for anti-cancer efficacy.	[144]
29	Cucurbitacin B	Autophagy	Approximately 1 µM	-	-	In vitro study Cervical cancer cell line and breast cancer cell lines	Cucurbitacin B induced caspase independent autophagic cell death in HeLa cells at 1 µM. Additionally, it induced autophagy and increased ROS levels in MCF-7 at 200 nM. Autophagic cell death in normal counterparts was not studied.	[104]
30	Cucurbitacin B	Apoptosis	0.2 µM	-	-	In vitro study Prostate cancer cell lines	Cucurbitacin B induced cell death and ROS production in human prostate cancer cell lines LNCaP and PC-3 at 0.2 µM while no significant cell death or ROS production was observed in normal prostate cell line PrEC.	[145]
31	Cucurbitacin B (Pharmacokinetic study)	-	-	2–4 mg/kg	C_max_: 9–31 µg/L	Animal subjects—Rats	Following an oral administration of 2–4 mg/kg, a significantly low plasma level was achieved in rat plasma than required for anti-cancer activity.	[146]
32	Silibinin	Autophagy	40 µM	-	-	In vitro study Fibrosarcoma cells	Silibinin induced autophagy in human fibrosarcoma cell lines HT1080 at 40 µM within a period of 4 h. Cellular death was concluded to be an autophagy mediated apoptosis process. Effect on normal cells was not studied.	[106]
33	Silibinin (Pharmacokinetic study)	-	-	360, 720 and 1440 mg	C_max_: 0.4, 1.4 and 4 ± 5.3 µM respectively	Human Subjects—Colorectal cancer patients	Silibinin formulated capsules showed considerably high levels required to exert its anti-tumor effect.	[147]
34	Allicin	Autophagy and apoptosis	15–30 µg/mL	-	-	In vitro study Non-small cell lung cancer cell lines	Allicin induced both autophagy and apoptosis in human lung cancer cell lines A549 and NCI-H460.	[109]
35	Carnosol	Autophagy	<25 µM	-	-	In vitro study Breast cancer cell lines	Carnosol induced ROS-mediated autophagy in triple negative breast cancer cell line MDA-MB-231. Effect on normal cell counterparts was not studied.	[113]
36	Quercetin	Autophagy	200 µM	100 mg/kg	-	In vitro study Osteosarcoma cell line Animal subjects—Mice	Quercetin induced ROS-mediated autophagic cell death in human osteosarcoma cell line MG-63 and also inhibited tumor growth in mice model.	[116]
37	Berberine	Autophagy	-	-	-	In vitro study Renal carcinoma cell lines and normal kidney cell line.	Berberine induced autophagic cell death in 786-O, ACHN cell lines via its photosensitizer activity when combined with laser. Additionally, it induced cell death in normal kidney cell line HK-2 at when combined with laser. Treatment with Berberine alone failed to induce cell death >20% in cancer cells, while it induced cell death >20% in HK-2 normal cells at 40 µM within 48 h.	[118]
38	Berberine (Pharmacokinetic study)	-	-	200 mg/kg	C_max_: 25.85 ± 7.34 µg/L	Animal subject— Rats	A plasma level of 25.85 ± 7.34 µg/L equivalent to 76 µM was achieved.	[148]
39	Nobiletin	Pyroptosis	34.85–35.31 µM	-	-	In vitro study Ovarian cancer cell lines	Nobiletin induced cytotoxicity at concentration of 35 µM but no data regarding its effect on normal cell lines were shown.	[120]
40	Nobiletin	Apoptosis	40–80 µM	100 mg/kg	-	In vitro study Ovarian cancer cell lines OVCAR-3 and A2780 Animal subject— Mice	Nobiletin exhibited cytotoxicity in ovarian cancer cell lines at 40–80 µM while the IC_50_ for normal ovarian cell line was around 160 µM. Additionally, significant tumor growth inhibition was observed in athymic nude mice model at a dose of 100 mg/kg.	[149]
41	Nobiletin (Pharmacokinetic study)	-	-	50 mg/kg	C_max_: 1.78 µg/mL in plasma	Animal subject—Rats	The plasma level achieved after oral administration is 1.78 µg/mL which correlates to approximately 4–5 µM.	[150]
42	Emodin	Anoikis	10 µM	-	-	In vitro study Gastric cancer cell lines	Significant difference observed in RhoA expression between cancer and normal cell lines.	[61]
43	Emodin	Apoptosis	70 µM	-	-	In vitro study Cancer cell lines isolated from breast, lung, colon, and cervix carcinomas, normal human fibroblasts, and normal human keratinocytes	No effect observed on normal cell lines after 48-h treatment while cytotoxicity was observed in cancer cell lines.	[151]
44	Emodin (Pharmacokinetic study)	-	-	4.5 mg/kg	C_max_: 0.2 ± 0.1 µM	Animal subject—Rats	The concentration of free emodin achieved in rat plasma after oral administration of rhubarb extract was found to be much lower than required for its anti-cancer activity.	[152]
